# Supplementary material for: Complexome profiling of the Chlamydomonas psb28 mutant reveals TEF5 as an early PSII assembly factor
Source: Plant Cell. 2025 Mar 17;37(6):koaf055. doi: 10.1093/plcell/koaf055 (PMC12164590; doi:10.1093/plcell/koaf055)
Supplement: koaf055_Supplementary_Data [file koaf055_supplementary_data.zip › psb28_interactive.zip!/plots/intensity/qualityPlots/peptides/ADH1_Cre17.g746997.t1.1.html]

### Cre17.g746997.t1.1

Trivial name: ADH1  
SeqCoverage [median]: 3.10  
Mass: 102.23 kDa  
  
 

| Sequence | MissedCleavage | Mass [Da] | wtIntensity | muIntensity | Score |
| --- | --- | --- | --- | --- | --- |
| AAAEAANAAR | 0 | 914.46 | 576.88 | 0.00 | 114.50 |
| AEAAAPVAAAPATPHAEVKK | 1 | 1899.02 | 424.61 | 0.00 | 47.68 |
|  |  |  | 1001.5 | 0.0 |  |
